# Pathology and mechanisms of cochlear aging

**DOI:** 10.1002/jnr.24439

**Published:** 2019-05-07

**Authors:** Elizabeth M. Keithley

**Affiliations:** ^1^ Division of Otolaryngology ‐ Head and Neck Surgery University of California San Diego California

**Keywords:** acoustic trauma, cochlear amplifier, cochlear hair cells, spiral ganglion, stria vascularis

## Abstract

Presbycusis, or age‐related hearing loss (ARHL), occurs in most mammals with variations in the age of onset, rate of decline, and magnitude of degeneration in the central nervous system and inner ear. The affected cochlear structures include the stria vascularis and its vasculature, spiral ligament, sensory hair cells and auditory neurons. Dysfunction of the stria vascularis results in a reduced endocochlear potential. Without this potential, the cochlear amplification provided by the electro‐motility of the outer hair cells is insufficient, and a high‐frequency hearing‐loss results. Degeneration of the sensory cells, especially the outer hair cells also leads to hearing loss due to lack of amplification. Neuronal degeneration, another hallmark of ARHL, most likely underlies difficulties with speech discrimination, especially in noisy environments. Noise exposure is a major cause of ARHL. It is well‐known to cause sensory cell degeneration, especially the outer hair cells at the high frequency end of the cochlea. Even loud, but not uncomfortable, sound levels can lead to synaptopathy and ultimately neuronal degeneration. Even in the absence of a noisy environment, aged cells degenerate. This pathology most likely results from damage to mitochondria and contributes to degenerative changes in the stria vascularis, hair cells, and neurons. The genetic underpinnings of ARHL are still unknown and most likely involve various combinations of genes. At present, the only effective strategy for reducing ARHL is prevention of noise exposure. If future strategies can improve mitochondrial activity and reduce oxidative damage in old age, these should also bring relief.

**Significance**

Presbycusis, the age‐related increase in hearing thresholds and increased difficulty in discriminating speech in noisy environments (ARHL), is the most common sensory deficit of aging in humans. Social isolation and cognitive decline accompany this disability. Establishing the etiology and biological mechanisms that underlie the pathology will drive any successful attempt to prevent or remediate this condition.

## 1 INTRODUCTION

The effects of the passage of time on the structure and function of the inner ear are certainly complex and extremely variable among species and individuals within each species, but in general, most mammals, especially humans, lose hearing sensitivity, more profoundly at high frequencies. Humans also lose the ability to discriminate speech in noisy environments. Variability in the age of onset and the magnitude of degenerative changes are major features of ARHL. Obviously, the interactions among underlying genetics and environmental exposures contribute to the phenotype that is observed at the end of life. This review will provide an overview of the pathology, genetic, metabolic and environmental variables known to play a role in cochlear aging.

## 2 PATHOLOGY

Degenerative changes in the inner ear of aged humans and other mammals occur among the sensory hair cells, the primary sensory neurons or spiral ganglion cells, and the cells of the stria vascularis and spiral ligament including the vasculature. The degenerative patterns of these structures were initially described in human temporal bones (Bredberg, 1968; Hawkins & Johnsson, 1968; Johnsson & Hawkins, 1972; Otte, Schuknecht, & Kerr, 1978; Schuknecht, 1974; Suga & Lindsay, 1976) and in the last 40 years many different species with varying lifespans, genetic mutations, and noise exposures have been examined (Altschuler et al., 2015; Engle, Tinling, & Recanzone, 2013; Gratton, Schmiedt, & Schulte, 1996; Gratton, Smyth, Lam, Boettcher, & Schmiedt, 1997; Keithley & Feldman, 1979, 1982; Keithley, Ryan, & Woolf, 1989; Ohlemiller, Dahl, & Gagnon, 2010; Sha et al., 2008) along with human (Kusunoki et al., 2004; Makary, Shin, Kujawa, Liberman, & Merchant, 2011; Viana et al., 2015; Wu et al., 2018). As in the human, there is a loss of the same cells in aged mammals. Different species and strains have different relative amounts of degeneration and time of onset.

### 2.1 Stria vascularis/spiral ligament

Although the condition of the spiral ligament, the stria vascularis and its vasculature are less frequently evaluated in aged cochleas, degeneration of these structures has been observed and measured in aged human temporal bones and represents a major pathology (Ishiyama, Tokita, Lopez, Tang, & Ishiyama, 2007; Kurata, Schachern, Paparella, & Cureoglu, 2016; Schuknecht, 1974; Schuknecht & Gacek, 1993; Suzuki et al., 2006). Quiet‐aged gerbil, rat, and beagle dog cochleas have strial and spiral ligament degeneration at both ends of the cochlear duct related to vascular degeneration in the same locations (Gratton et al., 1996,1997; Keithley, Ryan, & Feldman, 1992; Le & Keithley, 2007; Spicer & Schulte, 2002). *Sox10*, a transcription factor involved in the development and maintenance of cells derived from the neural crest, is reduced in cells of the stria vascularis and spiral ligament in aged CBA/CaJ mouse cochleas, as well as human temporal bones (Hao et al., 2014).

The consequence of this pathology, including reduced ATPase expression, is a reduction in the endocochlear potential (Gratton et al., 1996; Schmiedt, 2010; Schulte & Schmiedt, 1992). This potential is generated by the cells of the stria vascularis (Salt, Melichar, & Thalmann, 1987; Wangemann, 2006) and serves as the energy source for the conduction current and the cochlear amplifier. The hair‐cell conductance current is carried by potassium ions that are constantly recirculated back to the spiral ligament and stria vascularis after they pass through the hair cells, and then, back into the endolymphatic space where the cycle begins again (Schulte & Steel, 1994; Spicer & Schulte, 1991, 1996). The current drives the electro‐motility of the outer hair cells (Ashmore & Gale, 2004). This electro‐motility serves as an amplifier that is required for the perception of high‐frequency (>2 kHz) sounds, as it provides 50–70 dB of gain in the basal turn of the cochlea, the high‐frequency region, and approximately 20 dB in the apical turn, the low‐frequency region (see Schmiedt, 2010 for review). The loss of the conduction current and the endocochlear potential has the greatest effect on high‐frequency hearing, because of the reduced amplification, which explains the very common increase in hearing thresholds above 1–2 kHz seen in aged, human audiograms in non‐noise exposed humans (Dubno, Eckert, Lee, Matthews, & Schmiedt, 2013; Lang et al., 2010; Schmiedt, 2010). Dubno et al. (2013) used audiograms from animal studies, where the pathology could be accurately specified, to classify pure‐tone audiograms from aged humans with known noise‐exposure and concluded that the loss of the endocochlear potential is the major cause of audiometric threshold shifts in humans, and therefore the major cochlear factor underlying ARHL.

### 2.2 Sensory hair cells

**Figure 1:**
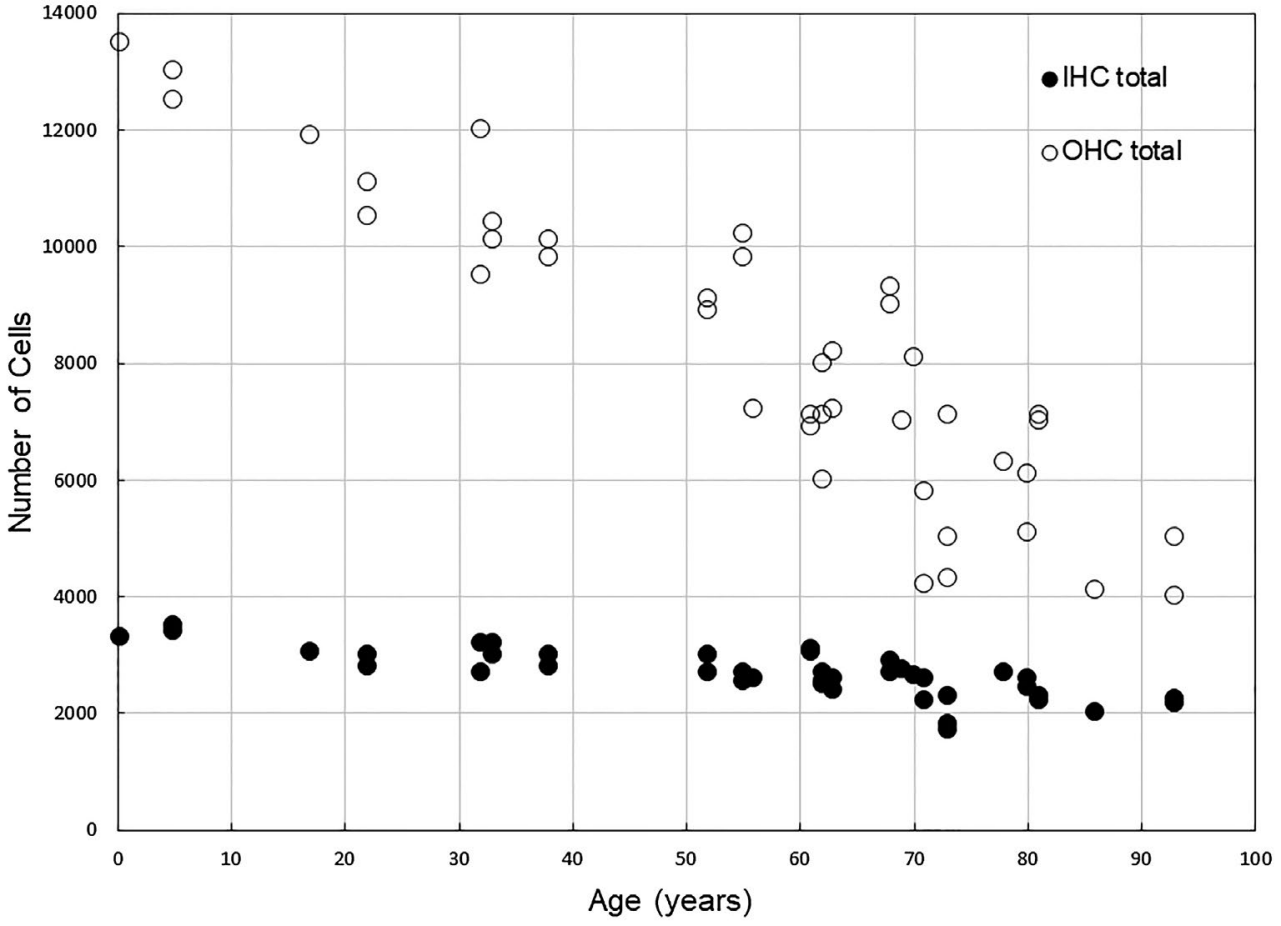
Number of total inner (closed circles) and outer hair cells (open circles) in human cochleas ranging in age from newborn to 93 years. The counts were made from surface preparations of human temporal bones by G. Bredberg (Figures 93 and 94, Bredberg, 1968). The loss of outer hair cells with age is greater than inner hair cells and starts early in life. Most cases used for this analysis were male and no information about noise exposure was given

When surface preparations of the human cochlea were developed, they enabled analysis of the whole sensory cell population. Bredberg (1968) prepared a detailed analysis of these cells from 125 cochleas from 78 individuals ranging from newborns to over 90 years of age. In addition to showing that the outer hair cells were more susceptible to degeneration with age (Figure 1), he documented that they degenerate in both the apical and basal cochlear turns. Because the common audiometric finding in aged humans is high frequency hearing loss, subsequent research has focused on the cochlear basal turn. The general perception, therefore, is that the loss of sensory cells in the basal turn is the major cochlear pathology of aging. It is also assumed that the difference between the human degenerative pattern and other mammals, except mice with the *ahl* gene (Johnson, Erway, Cook, Willott, & Zheng, 1997), is that the degeneration in humans occurs at the basal end of the cochlea, while in other mammals, the loss begins at the apical end and only later occurs in the basal turn. A more recent, quantitative study of the sensory cell loss in aged human cochleas, however, confirms the findings of Bredberg (1968). In humans, as in other mammals, the degeneration of sensory cells, especially the outer hair cells, begins in the apical and the basal turns and progresses throughout the length of the organ of Corti (Wu et al., 2018).

The consequence of the loss of outer hair cells and their electro‐motility in the basal turn is a loss of amplification for high‐frequency sounds, thus creating a hearing loss (Liberman & Kiang, 1978), similar to that observed with the loss of the endocochlear potential (Schmiedt, 2010). The loss of outer hair cells in the apical, low‐frequency portion of the organ of Corti does not have as large an impact on low‐frequency hearing thresholds because the electro‐motility amplification (20 dB) is not as large as it is for high‐frequency sounds. The effect of scattered loss of these cells on basilar membrane mechanics is not known. The loss of inner hair cells results in a loss of sensation at the specific frequency that was coded by those hair cells.

### 2.3 Auditory neurons/spiral ganglion cells

**Figure 2:**
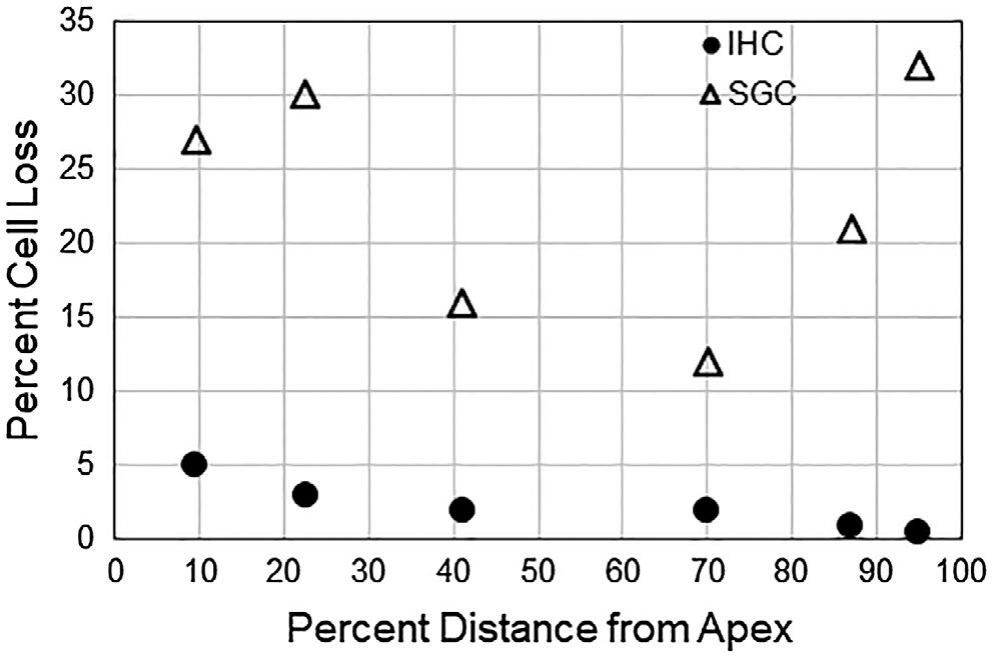
Comparison of the magnitude of inner hair cell loss (solid circles represent the median percent loss of 15 animals) and type‐I auditory neuronal loss (open triangles represent the median percent loss of 14 animals) in 27–34‐month‐old rat cochleas. The figure is redrawn from Keithley and Feldman (1982)

Neural degeneration is also a very common pathology of the aged inner ear, both in humans and other animals and occurs in both the apical and basal cochlear turns (Keithley & Feldman, 1979; Keithley et al., 1989; Makary et al., 2011; Otte et al., 1978; Viana et al., 2015). In fact, the magnitude of neuronal loss exceeds that of inner hair cell loss in both humans and other mammals (Chen, Webster, Yang, & Linthicum, 2006; Keithley & Feldman, 1982; Viana et al., 2015; Wu et al., 2018) (Figure 2). This is an important observation because it implies the neuronal degeneration is not simply secondary degeneration following the loss of inner hair cells with which they synapse. The loss of neurons does not result in elevated auditory brainstem response (ABR) thresholds, however, (Kujawa & Liberman, 2009; Schuknecht & Woellner, 1955), so likely does not affect audiometric thresholds in humans, and has therefore, been minimized in ideas of ARHL. Inadequate stimulus coding information to the central nervous system due to the loss of neurons, likely leads to difficulties in discrimination of sounds such as speech, especially in noise, a common problem of aged listeners.

## 3 MECHANISMS OF COCHLEAR CELL DEATH WITH AGE

### 3.1 Genetics

Given the many genetic causes of congenital and early onset hearing loss (Azaiez et al., 2018; Hereditary Hearing Loss website http://hereditaryhearingloss.org), it seems reasonable to expect that ARHL is also influenced by genetics. In fact, using the population of Framingham Heart Study participants, it was concluded that about 55% of the variance in auditory thresholds in aged siblings and parent/child relations was attributable to genetic causes (Gates, Couropmitree, & Myers, 1999). In spite of many researchers endeavoring to identify specific genes that underlie ARHL, no strong candidate has emerged yet. Several factors contribute to the difficulty of identifying one gene, and the first is, that probably multiple genes are involved, even in one individual. In addition, ARHL certainly results from various combinations of pathologies of the inner ear and central nervous system, each with a potentially different etiology and genetic contribution. Therefore, there are likely many genes and combinations of genes with subtle variants that make a person more, or less, susceptible to ARHL (Lewis et al., 2018), including genes that relate to susceptibility to acoustic trauma (Street et al., 2014). A genome‐wide‐association study (GWAS) of 100 inbred, mouse strains identified several gene loci that reached statistical significance and may, therefore, make an animal more vulnerable to damage from noise exposure (Lavinsky et al., 2016). These genes should also be examined in humans for their potential contribution to ARHL.

Studies of European and American‐aged populations have identified several chromosomal locations and genes related to hearing loss (Van Eyken, Camp, & Laer, 2007). Some, such as 11p, 11q13.5, and 14q, are near regions with known congenital, hearing‐loss genes (DeStefano, Gates, Heard‐Costa, Myers, & Baldwin, 2003) such as a region near the DFNA18 locus on chromosome 3q, which is related to a form of progressive, hereditary hearing loss (Garringer, Pankratz, Nichols, & Reed, 2006). A variant allele in the gene, *GRHL2*, a transcription factor on chromosome 8, that is related to DFNA28 has also been related to ARHL (Van Laer et al., 2008). Single nucleotide polymorphisms have been identified in genes coding anti‐oxidizing enzymes (Eyken, Camp, Fransen, et al., 2007). Huyghe et al. (2008) identified 8q24.13‐q24.22, a non‐coding region of chromosome 8, that is related to ARHL. Evaluation of aged, genetically heterogeneous mice revealed numerous loci on several chromosomes that are certainly related to ARHL (Schacht et al., 2012), though there does not seem to be any follow‐up on these leads. Evidence of genetic influence on cochlear physiology can be seen in mouse models with differing genetics. For instance, the endocochlear potential was measured in inbred mouse strains with various genetic backgrounds, and only four of the seven strains exhibited an age‐related decline in endocochlear potential, even at very old ages for mice (Ohlemiller, 2009). In addition, no one genotype was identified as having a propensity for strial degeneration.

A GWAS study of a European population identified many genes that may contribute to ARHL, but not one of them rose to statistical significance above the others (Fransen et al., 2015). These researchers concluded that genetic contributors to hearing loss are numerous and variable and that environmental factors are equal contributors to hearing loss in old age. The fact that the environment plays a large role seems very encouraging because it means that hearing loss is not an inevitable part of aging. Future studies of human genetics should attempt to exclude people with known noise exposure. It would also be valuable to follow people over time to establish relationships between the age of hearing‐loss onset and rate of decline relative to their genetic profile.

In a more recent report, Vuckovic at al. (2018) identified 21 potential genes related to ARHL and 2 genes, *CSMD1* and *PTRPD,* that might provide protection against ARHL. The specific function of the protein products of these genes in the inner ear is not known, though present in mouse, cochlear cDNA. The *PTRPD* gene codes for tyrosine phosphatase which is generally related to signaling molecules regulating several cellular processes, including cell growth and differentiation, activities that end in early postnatal life in the cochlea.

Yet another approach to identifying genes involved in ARHL is to use the ENU mutagenesis protocol to determine which mice have the phenotype of late‐onset hearing loss that looks like ARHL, and examine the genome for the mutation locus in those mice (Bowl & Brown, 2018; Potter et al., 2016). This is a straight‐forward strategy and with time will identify many, as yet unknown, genes that potentially participate in ARHL. The presence of similar mutations will then need to be identified in humans with ARHL. The initial report identified one gene*, Slc4a10*, localized to fibrocytes in the spiral ligament, whose protein product is known to be involved in sodium‐coupled bicarbonate transport regulating pH in cerebral spinal fluid as well as cytosolic fluid in neurons (NCBI database https://www.ncbi.nlm.nih.gov/gene/57282; Potter et al., 2016). If a similar gene mutation exists in aged humans, it could contribute to a phenotype of metabolic ARHL characterized by a gradually sloping hearing loss across frequencies (Dubno et al., 2013; Schmiedt, 2010). Not only does the ENU mutagenesis strategy provide new thoughts for mechanisms of ARHL, in this case, it also assists in understanding fluid regulation in the normal cochlea.

At this point, it is fair to say that the identification of genes that make an individual more susceptible to ARHL is a work in progress. There are clearly many genes that contribute to healthy functioning of the inner ear and as genetic analytic techniques improve, a clearer picture of which genes enable the observed pathology will emerge. Until then, localizing the many different, currently identified gene products in animal cochleas will help determine how they might affect hearing.

### 3.2 Metabolism/mitochondrial activity and mitochondrial genes

Energy, provided through cellular respiration in the mitochondria, is required for cell survival, and the decreased ability to provide sufficient energy, through oxidative phosphorylation, is certainly a potential mechanism of cell loss in all tissues, especially the energy‐demanding cochlea (Madreiter‐Sokolowski, Sokolowski, Waldeck‐Weiermair, Malli, & Graier, 2018; Pickles, 2004). It seems likely that during the production of adenosine triphosphate, reactive oxygen species (ROS) are generated and these have the capability of damaging DNA leading to loss of proteins and apoptosis (Ames, 2004; Lee & Wei, 2012). There are also anti‐oxidant enzymes that prevent the oxidizing damage, such as superoxide dismutase (SOD), catalase, glutathione peroxidase (GPX) and glutathione reductase and the balance between ROS and anti‐oxidants prevents cell death (Valko et al., 2007). Another cell‐death hypothesis is that mitochondria, in addition to cellular respiration, regulate life span and the aging process through signaling pathways involving insulin and other mediators (Bratic & Larsson, 2013). Decline in this function exacerbates aging. Little is known about insulin activity in the cochlea except caloric restriction and diabetes do affect hearing (Fowler et al., 2015). Both these ideas indicate that tissues with high energy requirements are vulnerable to aging.

The inner ear uses energy to maintain the endocochlear potential, generated by the stria vascularis, to assist motility in outer hair cells, perform synaptic activity, and to maintain the spontaneous and sound‐driven discharges of the auditory neurons in the spiral ganglion. The cells of the stria vascularis, the hair cells and the neurons all contain high concentrations of mitochondria (Nakazawa, Spicer, & Schulte, 1995; Spicer & Schulte, 2002; Spoendlin, 1981) and Na/K‐ATPase (Ding, Walton, Zhu, & Frisina, 2018; Ryan & Watts, 1991; Schulte & Adams, 1989).

Examination of the stria vascularis and spiral ligament in aged gerbils showed a decrease in the specific activity of Na/K‐ATPase in these tissues (Gratton et al., 1997), as well as degeneration of strial capillaries at both ends of the cochlear spiral (Gratton et al., 1996; Gratton & Schulte, 1995) and decreased blood flow (Prazma et al., 1990). In aged CBA/CaJ mice, Na/K‐ATPase expression was reduced by as much as 80%, and the stria vascularis was atrophied (Ding et al., 2018). It is difficult to determine whether the lack of blood flow or the cellular dysfunction leads to strial atrophy, so this question still needs to be addressed experimentally. Noise‐exposed hair cells also show ultrastructural signs of metabolic stress (Gannouni et al., 2015). Whether or not this contributes to cell death is unknown.

Since ROS have the capacity to damage mtDNA, celloidin sections from different temporal bone collections have been used to examine mtDNA deletions and mutations in aged humans. Bai, Seidman, Hinojosa, and Quirk (1997) found that 82% of 17 aged cases with hearing loss and 47% of those with no hearing loss had the common 4,977 base pair mtDNA deletion. Markaryan, Nelson, and Hinojosa (2009) also evaluated the common deletion in 19 ARHL cases and reported a relationship between the presence of the deletion and the magnitude of hearing loss. Likewise, Fischel‐Ghodsian et al., (1997) evaluated samples from 5 individuals with ARHL and found that numerous small deletions were present throughout the mtDNA. In a more recent study, using tissue samples from 400 individuals and the Affymetrix Human Mitochondrial Resequencing Array, Bonneux et al. (2011) found no relationship between the amount of mtDNA variants and hearing loss, although they did find many variants.

In an effort to determine whether mtDNA mutations, by themselves, can generate enough cellular damage to cause hearing loss, mice bred without the nuclear coded, mtDNA repair gene, polymerase gamma, *Polg*, (Kujoth et al., 2005) were examined and found to have severe ARHL and auditory neuronal loss (Crawley & Keithley, 2011; Niu, Trifunovic, Larsson, & Canlon, 2007; Someya et al., 2008; Yamasoba et al., 2007). Although this observation does not demonstrate that mtDNA damage is the cause of cochlear damage, it does demonstrate that, if enough damage occurs, it will affect cell survival and hearing, so it provides motivation to establish what level of mtDNA damage does cause hearing loss. Surprisingly, the stria vascularis was not as affected as the neurons, implying that other mechanisms, such as the condition of the vasculature, are yet to be defined.

The presence of mitochondrial enzymes that are part of the respiratory chain, such as cytochrome oxidase (COX), have been assessed using immunohistochemistry as a measure of mitochondrial “fitness.” Decreased immuno‐label, an imprecise measure of protein concentration, was seen in temporal bone sections from aged individuals, though the decrease did not correlate with the amount of hearing loss, nor with the number of remaining ganglion cells in the temporal bone (Keithley, Harris, Desai, Linthicum, & Fischel‐Ghodsian, 2001). On the contrary, Markaryan, Nelson, and Hinojosa (2010) did find a relationship between COX3 immunostaining of auditory neurons and ARHL in 3 cases. MtDNA damage has also been found in aged rat cochlear tissue (Seidman, Bai, Khan, & Quirk, 1997). Since decreased activity among auditory neurons is not reflected in threshold measures (Schuknecht & Woellner, 1955), a better measure in animal studies might be the spontaneous discharge rate profile of the auditory nerve fibers in old versus young animals, although this requires single unit physiology. The issue of the relationship between mtDNA damage and cell function in the stria vascularis, hair cells, and auditory neurons is not resolved and deserves more experimental examination.

Mouse models have also been developed to test whether an inadequate amount or overabundance of anti‐oxidant enzymes has an effect on ARHL or cell survival. The absence of SOD1 (Cu/Zn superoxide dismutase) resulted in a larger loss of hearing in aged mice than the background strain and a greater loss of auditory neurons and strial cells (Keithley et al., 2005; McFadden et al., 1999). GPX1 deficiency also results in increased hearing loss, hair cell, and neuronal loss (Ohlemiller, McFadden, Ding, Lear, & Ho, 2000). Over‐expression of catalase reduced ARHL and the loss of hair cells (Someya et al., 2009), while over‐abundance of SOD1 had little or no effect on ARHL (Keithley et al., 2005) or noise exposure in younger animals (Coling et al., 2003). Reduced amounts of manganese superoxide dismutase (SOD2), though naturally occurring in aged CBA/J mice (Jiang, Talaska, Schacht, & Sha, 2007) do not affect the cochlea (Kinoshita, Sakamoto, Kashio, Shimizu, & Yamasoba, 2013; Le & Keithley, 2007). In addition to transgenic mouse models, several groups have used Affymetrix gene chips (Affymetrix Inc., Santa Clara, CA) to examine gene products that work as anti‐oxidants to either reduce or oxidize specific proteins and found changes in expression levels with age (Tadros, D'Souza, Zhu, & Frisina, 2014). SOD2 promoter variants may also be related to ARHL in men (Nolan, Cadge, Gomez‐Dorado, & Dawson, 2013). These results are hard to interpret until more is known about specific antioxidant activity in the various cochlear cells. While there are many experiments that point to mitochondrial dysfunction contributing to age‐related cellular degeneration, it seems that better assays are needed to directly test the function of these organelles in aged cells.

### 3.3 Noise

The large loss of hair cells, especially the outer hair cells, at the basal end of the human cochlea is most likely the result of acoustic trauma (Johnsson & Hawkins, 1976; Liberman & Kiang, 1978), not healthy aging. The mechanism for hair cell death at the apex is not known. Many years of noise‐exposure experiments have detailed the levels and duration of noise that lead to cochlear cell degeneration. Aged animals raised in quiet environments do have outer hair cell losses in the basal turn, but generally of much less magnitude. The inner hair cell loss in these animals is very small (Keithley & Feldman, 1982; Liberman, 1978; Tarnowski, Schmiedt, Hellstrom, Lee, & Adams, 1991) making it clear that the life‐time noise exposure is an important variable in studies of ARHL.

Recent experiments indicate that even noise at a loud, but still “comfortable” level may be more damaging than previously thought. Young mice (4–16 weeks old) and guinea pigs, of similar age, exposed to 100 dB octave‐band noise, a level that causes only temporary threshold shifts, experience unexpected amounts of hearing loss at old ages. The hearing loss is accompanied by synaptic damage (synaptopathy) at the inner hair cell‐spiral ganglion cell ribbon synapse that leads to loss of the peripheral processes and neuronal cell bodies in the absence of inner hair cell degeneration (Kujawa & Liberman, 2006, 2009; Lin, Furman, Kujawa, & Liberman, 2011; Sergeyenko, Lall, Liberman, & Kujawa, 2013; Valero et al., 2017). The low‐spontaneous‐discharge‐rate fibers of the auditory neurons are more severely damaged than the medium and high spontaneous‐rate fibers (Furman, Kujawa, & Liberman, 2013) which is consistent with previous studies showing that low‐spontaneous rate fibers are more vulnerable to noise (Liberman, 1978) and aging (Schmiedt, 1996). It should be noted, however, that even aged, quiet reared gerbils experience a loss of radial fibers in the osseous spiral lamina (Suryadevara, Schulte, Schmiedt, & Slepecky, 2001). There is also a loss of outer hair cells from this 100 dB exposure (Fernandez, Jeffers, Lall, Liberman, & Kujawa, 2015). Chronic (6 hr/day for 3 months) exposure to the same octave‐band noise at even 70 or 85 dB caused neuronal degeneration in the absence of hair cell loss in Wistar rats that were examined at the end of the exposure period (Gannouni et al., 2015).

While these experimental data clearly demonstrate this phenomenon, called hidden hearing loss (Schaette & McAlpine, 2011), in laboratory animals, there is some controversy as to whether the same process occurs in humans (Guest, Munro, Prendergast, Millman, Plack, 2018; Prendergast et al., 2018). Because audiometric tests of pure‐tone thresholds do not provide a good measure of surviving neurons, the N_1_ peak of an ABR to click stimuli in animals was used to provide a more useful estimate (Prendergast et al., 2018). However, this measure in a large sample of relatively young humans (age 19–34 years) did not correlate with their memory of noise exposure in their youth. Similar studies with aged humans might reveal a correlation with early noise exposure, however such studies will have even greater difficulties is estimating early noise exposure. A non‐invasive method for estimating synaptic degeneration in humans might also help resolve the controversy (Bramhall, McMillan, Kujawa, & Konrad‐Martin, 2018).

In spite of the controversy, it seems that the experimental data in laboratory mammals, coupled with the human temporal bone data that show loss of radial fibers, and a greater loss of neurons than inner hair cells (Viana et al., 2015; Wu et al., 2018), support the interpretation that noise exposure at an early age of cochlear development has the potential to damage the inner‐hair cell synapse. It is interesting that both the mouse, with an altricial cochlea, and the guinea pig, with a precocial cochlea, as in the human, have a similar period of sensitivity post‐partum. The mouse cochlea is not functional until 8–9 days after birth while the human cochlea is fully functional at 18–20 weeks of gestation. Establishing precise critical periods of sensitivity might make correlating experimental and human results easier, although the problem of establishing audiometric tests and assessing human noise exposures still remains.

### 3.4 Ototoxic pharmaceuticals

Exposure to ototoxic drugs, especially chemotherapeutic agents and aminoglycoside antibiotics (Jiang, Karasawa, & Steyger, 2017; Steyger, Cunningham, Esquivel, Watts, & Zuo, 2018) leads to cochlear degeneration and hearing loss, but should be considered separate from ARHL when considering its mechanisms.

### 3.5 Inflammation

There has been little attention paid to immune responses in aged cochleas, although in the central nervous system, it is postulated that immune function can have a negative effect on neurons (Patterson, 2015). In the cochlea, immune‐surveillance occurs constantly (Hashimoto, Billings, Harris, Firestein, & Keithley, 2005) and noise exposure affects the capillary‐perivascular units of the stria vascularis (Shi, 2016) and stimulates the recruitment of circulating immunocytes to the cochlea (Hirose, Discolo, Keasler, & Ransohoff, 2005; Tornabene, Sato, Pham, Billings, & Keithley, 2006). These cells enter the cochlea through the post‐capillary venules in the spiral ligament, whose fibrocytes function to recirculate potassium and maintain the endocochlear potential. Whether the infiltration of cells and accompanying cytokine and chemokine expression has any effect on spiral ligament function is unknown, but Zhang, Liu, Soukup, and He (2014) suggest that this expression may be related to degenerative changes in these structures with age (Kusunoki et al., 2004). Because numerous pharmaceutical agents are available to reduce inflammation, and thereby protect the inner ear, more research into inner ear inflammatory activity during the lifetime should produce clinically relevant information.

## 4 STRATEGIES FOR MINIMIZING PRESBYCUSIS

Diet and exercise are often discussed in relation to healthy aging and the condition of the inner ear is no exception. Caloric restriction, long known to prolong life, beginning at a young age was effective in reducing hearing loss and cell degeneration in aged rats and mice (Seidman, 2000; Someya, Yamasoba, Weindruch, Prolla, & Tanokura, 2007). In collaboration with a larger study investigating the effects of a high anti‐oxidant diet on the central nervous system of beagle dogs (Fahnestock et al., 2012), Le and Keithley (2007) examined the cochleas from the same dogs reporting that there was a small decrease in the magnitude of auditory neuron degeneration and strial atrophy in the dogs that received anti‐oxidant supplementation. These studies, though small, support the idea that mitochondrial dysfunction contributes to cell dysfunction and death in aged cochlear cells and a diet high in antioxidants may reduce the pathology. Exercise also seems to reduce cochlear degeneration, including vascular degeneration in the stria vascularis, and hearing loss in mice given the opportunity to use a running wheel in their cage throughout their lifespan (Han et al., 2016).

## 5 CONCLUSIONS

ARHL of cochlear origin occurs in most mammalian species, with differences in the time of onset and the magnitude of loss among species. It is associated with degenerative changes including the highly energetic cells of the stria vascularis, the vasculature, auditory neurons, and sensory cells, especially the outer hair cells. The major factor contributing to increased auditory thresholds, in the absence of chronic noise‐induced damage, is the loss of the endocochlear potential (Dubno et al., 2013; Mills, Schmiedt, Schulte, & Dubno, 2006; Schmiedt, 2010) and the inability to understand speech‐in‐noise is likely related to neuronal degeneration (Liberman & Kujawa, 2017; Parthasarathy & Kujawa, 2018).

While genetic causes of cellular degeneration in the cochlea have not been identified, they certainly contribute to the aging phenotype (Gates et al., 1999), and while one gene will not be the cause, various combinations certainly will increase the propensity. Reduced mitochondrial function within the cells of the stria vascularis and auditory neurons has been assessed by many groups using different assays (Pickles, 2004). These include measures of mtDNA damage, effects of anti‐oxidants on cell survival and function, and the presence and activity of Na, K‐ATPase and other enzymes. The magnitude of the endocochlear potential is one of the best functional tests of the metabolic activity of the stria vascularis and this is decreased in aged gerbils and some mouse strains. Eating a healthy diet, rich in anti‐oxidants and exercising should contribute to protecting the stria vascularis and neurons.

The magnitude of ARHL could best be reduced by protection from noise exposure that directly leads to outer hair cell degeneration (Liberman & Kiang, 1978) and ultimately neuronal degeneration (Kujawa & Liberman, 2006) and inflammation (Hirose et al., 2005; Tornabene et al., 2006). Though not reviewed here, several groups are working on therapeutic, pharmaceutical strategies for addressing age‐related cochlear degeneration (Bielefeld, Tanaka, Chen, & Henderson, 2010; Frisina, Ding, Zhu, & Walton, 2016; Kalinec, Lomber, Urrutia, & Kalinec, 2017; Seidman, 2000; Seidman, Khan, Tang, & Quirk, 2002; Wan & Corfas, 2015) and the future might then see less debilitation from ARHL.

## CONFLICT OF INTEREST

The author has no conflicts of interest to declare.

## Supporting information

[Correction added on July 30, 2019 after first online publication: The heading ‘Transparent Peer Review Report.’ was added and Peer review communication document was uploaded online.]

Transparent Peer Review Report.
