# Supplementary material for: Pathology and mechanisms of cochlear aging
Source: J Neurosci Res. 2019 May 7;98(9):1674–84. doi: 10.1002/jnr.24439 (PMC7496655; doi:10.1002/jnr.24439)
Supplement: Supplementary file 1 — Transparent Peer Review Report. [file JNR-98-1674-s001.docx]

Pathology and Mechanisms of Cochlear Aging

Elizabeth M Keithley

_____________________________________________________________________________________

| Review timeline: | Submission date: 28/01/19  Editorial Decision: Minor Revision  Revision Received: 22/03/19  Accepted: 23/4/2019 |  |
| --- | --- | --- |
|  |  |  |

Editor: Eric Prager

Reviewer 1: Bradley Schulte

Reviewer 2: Federico Kalinec

Reviewer 3: Anne Giersch

_____________________________________________________________________________________

1st Editorial Decision	29/05/18

_____________________________________________________________________________________

Dear Dr Keithley: 

Thank you for submitting your manuscript to the Journal of Neuroscience Research. We've now received the reviewer feedback and have appended those reviews below. I'm glad to say that the reviewers are overall very enthusiastic and supportive of the review. They did raise some concerns and made some suggestions for clarification, but I expect that these points should be relatively straightforward to address. If there are any questions or points that are problematic, please feel free to contact me. I am glad to discuss. 

We ask that you return your manuscript within 30 days. Please explain in your cover letter how you have changed the present version. If you require longer than 30 days to make the revisions, please contact Dr. Eric Prager (eprager@wiley.com). You can submit your revised manuscript directly by clicking on the following link: 

*** PLEASE NOTE: This is a two-step process. After clicking on the link, you will be directed to a webpage to confirm. *** 

https://mc.manuscriptcentral.com/jnr?URL_MASK=85fb2dac0834406b9008dbf28ffa8ac8 

Thank you again for your submission to the Journal of Neuroscience Research; we look forward to reading your revised manuscript. 

Best Wishes, 

Dr Lisa Nolan 
Associate Editor, Journal of Neuroscience Research 

Dr Eric Prager 
Editor-in-Chief, Journal of Neuroscience Research 


Associate Editor: Nolan, Lisa 
Comments to the Author: 
This is a well-written mini-review discussing our current understanding of the pathology and mechanisms of cochlear ageing. Recent advances in the field are nicely discussed alongside the more historical studies. Insight is gained from both animal models of age-related hearing loss and genetic studies in humans. The review is very well-organised and the sections flow nicely. I enjoyed reading it. I have a couple of general points and some more minor comments. 

General points 
• Please use one term throughout the text: age-related hearing loss or presbycusis. 
• When discussing genes identified from genome wide association studies for ARHL/adult hearing status please clarify if these genes reached genome wide significance or not. 

Comments by page 
Page 3, line 34: 
Text: “…there is a loss of cells in aged mammals.” 
Comment: Which cells? 

Page 3, line 46: 
Text: “…..and may be the major pathology…” 
Comment: I am not clear if you mean “degeneration” of the spiral ligament/stria vascularis “may be the major pathology” or if you mean these cochlear sub-regions “may be the major pathology”? The 2006 paper by Suzuki et al only focused on quantitative changes in the spiral ligament/stria vascularis and did not examine any other cochlear sub-regions. 

Page 4, line 2: 
This sentence is quite long and the text at the end is not very clear: “…..energy source for the hair-cell conduction current and the cochlear amplifier, the electro-motility of the outer hair cells”. Two shorter sentences may be more helpful. 

Page 4, line 12: 
A Reference is missing? 

Page 4, line 37: 
The reference for Bredberg is missing. 

Page 4, line 41: 
This sentence would benefit from being split into 2 smaller sentences. 

Page 4, line 54 
Text: “….non-human mammals….” 
Comment: please clarify. 

Page 5,line 44 
Text: “……many genetic causes of hearing loss….” 
Comment: Please clarify you mean congenital/early onset hearing loss. 

Page 6, line 4 
Text: “….genetic linkage…” 
Comment: I am not clear what you mean by use of the terminology genetic linkage in this context? Use of the following may be more helpful: “…..genetic landscape / genetic architecture….” 

Page 6, line 9 
Please write GWAS in full at first use and then abbreviate. 

Page 6, line 46 
Text: “….identified the GMR7 gene as having more SNPs than other genes in the hearing loss cohort….” 
Comment: This sentence is not very clear. 

Page 7, line 11 
With regards to the 2018 study by Vuckovic et al, genetic variants in CSMD1 and PTRPD were enriched in the control population, not the subjects with ARHL. Hence, suggesting a protective role of these genes. Please amend. 

Page 7, line 22 
A reference is missing for this statement. 

Page 7, line 27 
Text: “….evaluate the genome….” 
Suggest: “…..examine the genome 

Page 9, line 12 
Text: “….the amount of deletions and hearing loss…..” 
Comment: This study was not limited to the detection of mtDNA deletions, but rather mtDNA variants. Please revise terminology used. 

Page 9, line 34 
Please clarify: “……decreased label…..” 

Page 10, line 9 
Regarding the discussion on SOD2, the 2013 study by Nolan et al might be of interest here: Mech Ageing Dev. 2013 Jul-Aug;134(7-8):298-306. doi: 10.1016/j.mad.2013.02.009. Epub 2013 Mar 13. 

Page 12, line 56 
Text: “The major factor contributing to increased auditory thresholds, in the absence of chronic noise-induced damage, is the loss of the endocochlear potential” 
Comment: I find this sentence quite bold – especially since we cannot directly measure the EP in humans. 

Page 13, line 22 
Text: “….degeneration (Liberman & Kiang, 1978), ultimately neuronal degeneration….” 
Comment: the word “and” would appear to be missing? 

Reviewer: 1 

Comments to the Author 
Age-related hearing loss (ARHL), also known as presbycusis, is the most prevalent form of hearing loss in humans. In aging adults of both genders the decreased hearing sensitivity, decreased ability to understand speech in a noisy environment, and impaired sound localization, is not only evident, but interferes with their social interactions, leading to withdrawal, depression, inability to work and increased economic hardship for the all family. However, to date, there are no clinical strategies to prevent and/or ameliorate ARHL. 
ARHL is associated to a combination of environmental and genetic factors, further complicated by association with other forms of age-related morbidity including cardiovascular diseases and cancer. Therefore, reviews providing updated overviews of the genetic, metabolic, and environmental factors known to play a role in cochlear aging are always welcome by researchers working in the hearing field. 
This review by Elizabeth Keithley is generally well written and provides useful information, particularly on the genetic and metabolic changes associated with ARHL. However, I think the manuscript needs some grammatical editing. Among others, the author should pay attention to the following points: 

1- Abstract, page 2, line 7. 
Say: “The affected cells in the cochlea include the stria vascularis and its vasculature, spiral ligament, ….” 
Suggestion: “The affected areas (or regions) in the cochlea include the stria vascularis and its vasculature, spiral ligament, ….” 
or 
“The affected cells in the cochlea include those of the stria vascularis and its vasculature, spiral ligament, ….” 

2- Introduction, page 3, lines 5-7 
Say: “…, but in general most mammals, specially humans, lose hearing sensitivity, most profoundly at high frequencies.” 
Suggestion: “…, but most mammals, specially humans, lose hearing sensitivity more profoundly at high frequencies.” 

3- Introduction, page 3, lines 13-14 
Say: “… contribute to the phenotype that is evaluated at the end of life.” 
Suggestion: “… contribute to the phenotype observed at the end of life.” 

4- Pathology, page 3, lines 22-23 
Say: “… the sensory hair cells, the primary sensory neurons or spiral ganglion cells and the cells of the stria vascularis and the …” 
Suggestion: “… the sensory hair cells, the primary sensory neurons or spiral ganglion cells, and the cells of the stria vascularis and the …” 

5- Pathology, page 4, lines 5-6 
Say: “… and serves as the battery or energy source for the hair cell …” 
Suggestion: “… and serves as the energy source for the hair cell …” 

6- Pathology, page 4, lines 7-16 
Say: “The amplifier is…. The hair-cell conductance current is …” 
Suggestion (change order): “The hair-cell conductance current is … The amplifier is….” 

7- Sensory Hair Cells, page 4, lines 40-51 
Say: “Because the … in the basal turn” 
Suggestion: The sentence is too long. It should be divided in, at least, 2 shorter sentences. 

8- Sensory Hair Cells, page 5, lines 1-14 
Say: “The consequence of inner hair cell loss …The loss of outer hair cells …” 
Suggestion (change order): “The loss of outer hair cells … The consequence of inner hair cell loss …” 

9-  Genetics, page 5, line 49 
Say: “… that underlie age-related hearing loss, however, at this time, no strong candidate has emerged” 
Suggestion: “… that underlie age-related hearing loss, but no strong candidate has emerged yet” 

10-  Genetics, page 6, line 54 
Say: “A GWAS study, however, of another European population identified many genes…” 
Suggestion: “A GWAS study of another European population, however, identified many genes…” 

11-  Metabolism/, page 8, lines 12-16 
Suggestion: Eliminate the sentence “In fact, reduced …Prolla and Denu, 2014).” 


Reviewer: 2 

Comments to the Author 
This is a nice mini-review of age-related hearing loss. Due to it's nature, as a mini-review it is fairly superficial, but it does hit all the relevant issues.  I would like to see a bit more development of section 3.4 Ototoxic Drugs. Right now it is one sentence (!) and there are some very interesting genetics (such as mitochondrial mutations) that should be mentioned. Other than that, nice job. 

Reviewer: 3 

Comments to the Author 
This is an informative and well-written article that summarizes current knowledge about the cause(s) and ramifications of age-related hearing loss.  The potential causes of presbyacusis including both the intrinsic genetically controlled aging process and extrinsic accumulated environmental stresses, in particular noise exposure, are presented in a fair and balanced fashion.  Likewise, the pathological manifestations of genetic alterations, about which little is yet known, and environmental insults in the inner ear are reviewed in considerable detail given the abbreviated length of the paper.  The physiological consequences of the pathological alterations in the stria vascularis and lateral wall, primary auditory neurons and hair cells are described thoroughly, especially as they relate to cochlear amplification and speech perception.  The inclusion of some general concepts about aging such as reactive oxygen species toxicity and DNA and mitochondrial damage as they may relate to cochlear metabolism also is appreciated. 
Below I have provided a few key references that could be added to the review as well as some minor editorial suggestions. 

References: 
1) Schuknecht HF, Gacek MR (1993). Cochlear pathology in presbyacusis.  Ann Otol Rhino Laryngol 102, 1-16. PMID: 8420477.  This paper published towards the end of Dr. Schuknecht’s career, and based largely on studies of human temporal bones, concluded that atrophy of the stria vascularis is the predominant lesion in the aging ear. 
2) Schulte BA, Schmiedt RA (1992). Lateral wall Na,K-ATPase and endocochlear potentials decline with age in quiet-reared gerbils. Hear Res 61, 35-46. PMID: 1326508. This paper shows a direct relationship to the volume of the stria immunostained positively for Na,K-ATPase and the magnitude of the resting EP. 
3) Schulte BA, Steel KP (1994). Expression of alpha and beta subunit isoforms of Na,K-ATPase in the mouse inner ear and changes with mutations at the Wv or Sld loci. Hear Res 78, 65-76. PMID:7961179. This article is the first reference in the literature proposing that K+ is recycled from perilymph to endolymph via the lateral wall. 
4) Mills JH, Schmiedt RA, Schulte BA, Dubno JR (2006). Age-related hearing loss: A loss of voltage, not hair cells. Semin Hear 27, 228-236. DOI: 10.1055/s-2006-954849. This paper includes data showing the loss as well as shrinkage of spiral ganglion neurons in quiet-aged gerbils. 

Minor editorial suggestions: 
Page 2, line 7 – suggest changing this to read “The affected regions and cells” 
Page 2, lines 24-26 – sentence is unclear 
Page 3, line 43 – much less information relative to what? 
Page 3, line 48 – add Schucknecht and Gacek reference above 
Page 6, line 39 – suggest change to “an age-related decline in endocochlear” 
Page 6, line 47 – suggest substituting “associated with” for “certainly related to” 
Page 7, line 14-15 – meaning of sentence is unclear 
Page 7, line 47 – nuclear ?? 
Page 8, line 14 – insert “with age”

____________________________________________________________________________________

Authors’ Response	06/06/2018

_____________________________________________________________________________________

This is a well-written mini-review discussing our current understanding of the pathology and mechanisms of cochlear ageing. Recent advances in the field are nicely discussed alongside the more historical studies. Insight is gained from both animal models of age-related hearing loss and genetic studies in humans. The review is very well-organised and the sections flow nicely. I enjoyed reading it. I have a couple of general points and some more minor comments.

General points

• Please use one term throughout the text: age-related hearing loss or presbycusis. done

• When discussing genes identified from genome wide association studies for ARHL/adult hearing status please clarify if these genes reached genome wide significance or not. This comment led me to read about GWAS and statistics and as a result I have deleted the reference to GRM7 (Freidman et al 2009 and Newman et al 2012) because the Freidman et al paper did not have sufficient statistical significance.

Comments by page

Page 3, line 34:

Text: “…there is a loss of cells in aged mammals.”

Comment: Which cells? done

Page 3, line 46:

Text: “…..and may be the major pathology…”

Comment: I am not clear if you mean “degeneration” of the spiral ligament/stria vascularis “may be the major pathology” or if you mean these cochlear sub-regions “may be the major pathology”? The 2006 paper by Suzuki et al only focused on quantitative changes in the spiral ligament/stria vascularis and did not examine any other cochlear sub-regions. Done fair enough. I de-emphasized the strial pathology.

Page 4, line 2:

This sentence is quite long and the text at the end is not very clear: “…..energy source for the hair-cell conduction current and the cochlear amplifier, the electro-motility of the outer hair cells”. Two shorter sentences may be more helpful. I simplified the sentences, focusing on the cochlear amplifier function of the stria.

Page 4, line 12:

A Reference is missing? Added “see Schmiedt, 2010 for review”; the paragraph has been revised.

Page 4, line 37:

The reference for Bredberg is missing. The reference has been placed at the beginning of the sentence, rather than at the end.

Page 4, line 41:

This sentence would benefit from being split into 2 smaller sentences. This sentence has been shortened.

Page 4, line 54

Text: “….non-human mammals….”

Comment: please clarify. I changed the word to “other”.

Page 5,line 44

Text: “……many genetic causes of hearing loss….”

Comment: Please clarify you mean congenital/early onset hearing loss. These words were added to the sentence.

Page 6, line 4

Text: “….genetic linkage…”

Comment: I am not clear what you mean by use of the terminology genetic linkage in this context? Use of the following may be more helpful: “…..genetic landscape / genetic architecture….” I changed “linkage” to “contribution“

Page 6, line 9

Please write GWAS in full at first use and then abbreviate. “genome-wide-association study” was added

Page 6, line 46

Text: “….identified the GMR7 gene as having more SNPs than other genes in the hearing loss cohort….”

Comment: This sentence is not very clear. This sentence has been deleted because of earlier comment.

Page 7, line 11

With regards to the 2018 study by Vuckovic et al, genetic variants in CSMD1 and PTRPD were enriched in the control population, not the subjects with ARHL. Hence, suggesting a protective role of these genes. Please amend. This paragraph has been re-written. Thank you for identifying my misunderstanding.

Page 7, line 22

A reference is missing for this statement. Because the thought is purely speculative, the sentence has been deleted. The whole paragraph has been rewritten.

Page 7, line 27

Text: “….evaluate the genome….”

Suggest: “…..examine the genome. This change was made

Page 9, line 12

Text: “….the amount of deletions and hearing loss…..”

Comment: This study was not limited to the detection of mtDNA deletions, but rather mtDNA variants. Please revise terminology used. The terminology has been corrected.

Page 9, line 34

Please clarify: “……decreased label…..” a definition has been inserted.

Page 10, line 9

Regarding the discussion on SOD2, the 2013 study by Nolan et al might be of interest here: Mech Ageing Dev. 2013 Jul-Aug;134(7-8):298-306. doi: 10.1016/j.mad.2013.02.009. Epub 2013 Mar 13. A sentence has been added

Page 12, line 56

Text: “The major factor contributing to increased auditory thresholds, in the absence of chronic noise-induced damage, is the loss of the endocochlear potential”

Comment: I find this sentence quite bold – especially since we cannot directly measure the EP in humans. I thought I presented data throughout the manuscript to support this bold statement and I would like to keep it, if that is all right with you. I added another reference supporting the idea.

Page 13, line 22

Text: “….degeneration (Liberman & Kiang, 1978), ultimately neuronal degeneration….”

Comment: the word “and” would appear to be missing? An “and” was added.

----------------------------------------------------------------------------------

Reviewer: 1

Comments to the Author

Age-related hearing loss (ARHL), also known as presbycusis, is the most prevalent form of hearing loss in humans. In aging adults of both genders the decreased hearing sensitivity, decreased ability to understand speech in a noisy environment, and impaired sound localization, is not only evident, but interferes with their social interactions, leading to withdrawal, depression, inability to work and increased economic hardship for the all family. However, to date, there are no clinical strategies to prevent and/or ameliorate ARHL.

ARHL is associated to a combination of environmental and genetic factors, further complicated by association with other forms of age-related morbidity including cardiovascular diseases and cancer. Therefore, reviews providing updated overviews of the genetic, metabolic, and environmental factors known to play a role in cochlear aging are always welcome by researchers working in the hearing field.

This review by Elizabeth Keithley is generally well written and provides useful information, particularly on the genetic and metabolic changes associated with ARHL. However, I think the manuscript needs some grammatical editing. Among others, the author should pay attention to the following points:

1- Abstract, page 2, line 7. It now says “cochlear structures” rather than “cells” Say: “The affected cells in the cochlea include the stria vascularis and its vasculature, spiral ligament, ….”

Suggestion: “The affected areas (or regions) in the cochlea include the stria vascularis and its vasculature, spiral ligament, ….”

or

“The affected cells in the cochlea include those of the stria vascularis and its vasculature, spiral ligament, ….”

2- Introduction, page 3, lines 5-7 “most” has been changed to “more”

Say: “…, but in general most mammals, specially humans, lose hearing sensitivity, most profoundly at high frequencies.”

Suggestion: “…, but most mammals, specially humans, lose hearing sensitivity more profoundly at high frequencies.”

3- Introduction, page 3, lines 13-14 “evaluated“ has been changed to “observed”

Say: “… contribute to the phenotype that is evaluated at the end of life.”

Suggestion: “… contribute to the phenotype observed at the end of life.”

4- Pathology, page 3, lines 22-23 The comma has been added.

Say: “… the sensory hair cells, the primary sensory neurons or spiral ganglion cells and the cells of the stria vascularis and the …”

Suggestion: “… the sensory hair cells, the primary sensory neurons or spiral ganglion cells, and the cells of the stria vascularis and the …”

5- Pathology, page 4, lines 5-6 the word, “battery” was deleted

Say: “… and serves as the battery or energy source for the hair cell …”

Suggestion: “… and serves as the energy source for the hair cell …”

6- Pathology, page 4, lines 7-16 This paragraph has been modified according to the multiple suggestions

Say: “The amplifier is…. The hair-cell conductance current is …”

Suggestion (change order): “The hair-cell conductance current is … The amplifier is….”

7- Sensory Hair Cells, page 4, lines 40-51 The “and” was removed and now there are 2 sentences.

Say: “Because the … in the basal turn”

Suggestion: The sentence is too long. It should be divided in, at least, 2 shorter sentences.

8- Sensory Hair Cells, page 5, lines 1-14 The thoughts have been reversed

Say: “The consequence of inner hair cell loss …The loss of outer hair cells …”

Suggestion (change order): “The loss of outer hair cells … The consequence of inner hair cell loss …”

9- Genetics, page 5, line 49 This change was made.

Say: “… that underlie age-related hearing loss, however, at this time, no strong candidate has emerged”

Suggestion: “… that underlie age-related hearing loss, but no strong candidate has emerged yet”

10- Genetics, page 6, line 54 This change was made.

Say: “A GWAS study, however, of another European population identified many genes…”

Suggestion: “A GWAS study of another European population, however, identified many genes…”

11- Metabolism/, page 8, lines 12-16 This change was made.

Suggestion: Eliminate the sentence “In fact, reduced …Prolla and Denu, 2014).”

--------------------------------------------------------------------------------

Reviewer: 2

Comments to the Author

This is a nice mini-review of age-related hearing loss. Due to it's nature, as a mini-review it is fairly superficial, but it does hit all the relevant issues. I would like to see a bit more development of section 3.4 Ototoxic Drugs. Right now it is one sentence (!) and there are some very interesting genetics (such as mitochondrial mutations) that should be mentioned. Other than that, nice job. I disagree. Ototoxicity is a very important and complex topic, but this mini-review is not the place to discuss it. I only mentioned it for completeness.

----------------------------------------------------------------------------------------

Reviewer: 3

Comments to the Author

This is an informative and well-written article that summarizes current knowledge about the cause(s) and ramifications of age-related hearing loss. The potential causes of presbyacusis including both the intrinsic genetically controlled aging process and extrinsic accumulated environmental stresses, in particular noise exposure, are presented in a fair and balanced fashion. Likewise, the pathological manifestations of genetic alterations, about which little is yet known, and environmental insults in the inner ear are reviewed in considerable detail given the abbreviated length of the paper. The physiological consequences of the pathological alterations in the stria vascularis and lateral wall, primary auditory neurons and hair cells are described thoroughly, especially as they relate to cochlear amplification and speech perception. The inclusion of some general concepts about aging such as reactive oxygen species toxicity and DNA and mitochondrial damage as they may relate to cochlear metabolism also is appreciated.

Below I have provided a few key references that could be added to the review as well as some minor editorial suggestions.

References:

1) Schuknecht HF, Gacek MR (1993). Cochlear pathology in presbyacusis. Ann Otol Rhino Laryngol 102, 1-16. PMID: 8420477. This paper published towards the end of Dr. Schuknecht’s career, and based largely on studies of human temporal bones, concluded that atrophy of the stria vascularis is the predominant lesion in the aging ear.This reference has been added.

2) Schulte BA, Schmiedt RA (1992). Lateral wall Na,K-ATPase and endocochlear potentials decline with age in quiet-reared gerbils. Hear Res 61, 35-46. PMID: 1326508. This paper shows a direct relationship to the volume of the stria immunostained positively for Na,K-ATPase and the magnitude of the resting EP.This reference has been added.

3) Schulte BA, Steel KP (1994). Expression of alpha and beta subunit isoforms of Na,K-ATPase in the mouse inner ear and changes with mutations at the Wv or Sld loci. Hear Res 78, 65-76. PMID:7961179. This article is the first reference in the literature proposing that K+ is recycled from perilymph to endolymph via the lateral wall. This reference has been added.

4) Mills JH, Schmiedt RA, Schulte BA, Dubno JR (2006). Age-related hearing loss: A loss of voltage, not hair cells. Semin Hear 27, 228-236. DOI: 10.1055/s-2006-954849. This paper includes data showing the loss as well as shrinkage of spiral ganglion neurons in quiet-aged gerbils. This reference has been added.

Minor editorial suggestions:

Page 2, line 7 – suggest changing this to read “The affected regions and cells” changed

Page 2, lines 24-26 – sentence is unclear The sentence has been revised.

Page 3, line 43 – much less information relative to what? This sentence has been changed.

Page 3, line 48 – add Schucknecht and Gacek reference above Reference is added

Page 6, line 39 – suggest change to “an age-related decline in endocochlear” The wording was changed.

Page 6, line 47 – suggest substituting “associated with” for “certainly related to” wording is changed

Page 7, line 14-15 – meaning of sentence is unclear This whole paragraph has been re-written.

Page 7, line 47 – nuclear ?? The word “nuclear” has been deleted.

Page 8, line 14 – insert “with age” This sentence has been removed at the request of Reviewer #1
